# The Effectiveness and Adverse Events of Cannabidiol and Tetrahydrocannabinol Used in the Treatment of Anxiety Disorders in a PTSD Subpopulation: An Interim Analysis of an Observational Study

**DOI:** 10.1177/87551225231180796

**Published:** 2023-06-24

**Authors:** Sophie K. Stack, Nial J. Wheate, Niamh C. Moloney, Sarah V. Abelev, John W. Barlow, Elise A. Schubert

**Affiliations:** 1 School of Pharmacy, Faculty of Medicine and Health, The University of Sydney, Sydney, NSW, Australia; 2 Applied Cannabis Research, Sydney, NSW, Australia

**Keywords:** cannabis, anxiety, PTSD, cannabidiol, tetrahydrocannabinol, formulation

## Abstract

**Background:** Anxiety is a condition for which current treatments are often limited by adverse events (AEs). Components of medicinal cannabis, cannabidiol (CBD) and tetrahydrocannabinol (THC), have been proposed as potential treatments for anxiety disorders, specifically posttraumatic stress disorder (PTSD). **Objective:** To evaluate quality-of-life outcomes after treatment with various cannabis formulations to determine the effectiveness and associated AEs. **Methods:** An interim analysis of data collected between September 2018 and June 2021 from the CA Clinics Observational Study. Patient-Reported Outcomes Measurement Information System-29 survey scores of 198 participants with an anxiety disorder were compared at baseline and after treatment with medicinal cannabis. The data of 568 anxiety participants were also analyzed to examine the AEs they experienced by the Medical Dictionary for Regulatory Activities organ system class. **Results:** The median doses taken were 50.0 mg/day for CBD and 4.4 mg/day for THC. The total participant sample reported significantly improved anxiety, depression, fatigue, and ability to take part in social roles and activities. Those who were diagnosed with PTSD (n = 57) reported significantly improved anxiety, depression, fatigue, and social abilities. The most common AEs reported across the whole participant cohort were dry mouth (32.6%), somnolence (31.3%), and fatigue (18.5%), but incidence varied with different cannabis formulations. The inclusion of THC in a formulation was significantly associated with experiencing gastrointestinal AEs; specifically dry mouth and nausea. **Conclusions:** Formulations of cannabis significantly improved anxiety, depression, fatigue, and the ability to participate in social activities in participants with anxiety disorders. The AEs experienced by participants are consistent with those in other studies.

## Introduction

As defined by Craske and Stein,^1^ anxiety is a response that individuals have to situations they perceive as threatening, and anxiety disorders occur when this response is overactive to the point that it impairs a person’s functioning. The exact pathophysiology of anxiety is unknown; however, it is thought to arise from an imbalance of neurotransmitters such as serotonin and noradrenaline and abnormalities in brain structures including the amygdala and prefrontal cortex.^2^ The major types of anxiety disorders are generalized anxiety disorder, social anxiety, panic disorder, phobic disorder, posttraumatic stress disorder (PTSD), and obsessive compulsive disorder.^1^ The initial treatment for anxiety disorders is usually nonpharmaceutical, such as cognitive behavioral therapy, and when this is not suitable or successful, pharmacological treatment may be considered.^3,4^

Benzodiazepines are effective anxiolytics; however, they are limited to short-term or acute crisis use due to the risk of dependence, daytime sedation, and memory problems.^3,4^ The various antidepressants indicated for anxiety disorders, such as serotonin reuptake inhibitors, are only effective in 60% of people with PTSD and can be inconsistent in terms of efficacy.^5,6^ Due to the limited efficacy of some of these medications, clinicians take adverse event (AE) profiles and patient preference into consideration when prescribing these treatments.^3^ As such, there is a continuing need for new medications for anxiety disorders that have both favorable safety and efficacy profiles.

Cannabis is being explored as a treatment for a number of conditions including epilepsy, pain, and anxiety.^7^ The major cannabinoid constituents of cannabis are cannabidiol (CBD) and tetrahydrocannabinol (THC), which interact differently with the receptors of the endocannabinoid system, as well as other receptors in the body, and therefore have different therapeutic applications.^8^ It has been proposed that cannabinoids may be useful in the treatment of a number of anxiety disorders.^7,9-11^

The endocannabinoid system is distributed widely throughout the body and consists of cannabinoid type 1 receptors (CB1Rs) and cannabinoid type 2 receptors (CB2Rs).^12^ Cannabinoid type 1 receptors are more abundant in the central nervous system and brain, and CB2Rs are more widely distributed in the peripheral nervous system.^13^ Tetrahydrocannabinol interacts with CB1Rs in the brain, producing the associated anxiogenic effects of cannabis.^7^ High doses of THC are thought to increase the risk of anxiety exacerbations, whereas low doses, less than 30 mg of THC per day, could be an effective treatment for anxiety disorders.^14^ Cannabidiol has anxiolytic properties, and doses between 25 and 600 mg have been shown to decrease anxiety in participants.^15^ The exact mechanisms behind the anxiolytic properties of CBD are largely unknown; however, it does not interact with CB1Rs in the brain and is therefore not associated with anxiogenic effects at higher doses.^7^

Medicinal cannabis could be an effective treatment for a number of anxiety disorders, particularly PTSD; however, more research into the efficacy and AEs of CBD and THC is needed. In this article, we aimed to evaluate quality-of-life outcomes after treatment with various cannabis formulations to determine effectiveness and associated AEs in those taking medicinal cannabis for anxiety disorders.

## Methods

### Setting

We performed an interim analysis of data that had been collected from the CA Clinics Observational Study (CACOS), an ongoing, observational study conducted through the network of medicinal cannabis prescribers located in Australia. The purpose of CACOS is to collect data about the safety and efficacy of medicinal cannabis treatments in Australia using patient-reported outcome measures. The study includes a number of combined and parallel arms including but not limited to chronic pain (including endometriosis, fibromyalgia, and neuropathic pain), psychiatric conditions (including anxiety and PTSD), and neurological conditions (including epilepsy). Participants are enrolled before their first appointment and returned surveys that measured various health-related quality-of-life outcomes in participants.

### Participants

All participants that were enrolled in CACOS and using medicinal cannabis for an anxiety disorder were included in this analysis. Participants who took a baseline survey before treatment, and at least 1 survey after they had started treatment, were included in the Patient-Reported Outcomes Measurement Information System-29 (PROMIS-29) analysis. The PROMIS-29 questionnaire assesses pain intensity using a single 0-10 numeric rating item and 7 health domains (physical function, fatigue, pain interference, depressive symptoms, anxiety, ability to participate in social roles and activities, and sleep disturbance) using 4 items for each domain, to assess patient functioning and wellbeing. Anyone who returned at least 1 survey while taking medicinal cannabis were included in the AE analysis. The time between a participant’s first and last surveys defined their observational period, and where there was only 1 survey, the observational period was 1 day. These data were collected between the dates of the September 25, 2018, and June 29, 2021.

### Cannabis Formulations

The medicinal cannabis formulations that were prescribed to each participant contained either CBD or THC. Participants self-reported the medicinal cannabis product(s) they were taking, and based on the concentration of each constituent, they were classed into 1 of 5 groups: CBD- or THC-only, CBD- or THC-dominant, or balanced formulations. “Dominant” refers to a concentration of 1 constituent that is 1.5-times greater than the other. If the participants were switched to another formulation class during the observational period, they were included in both groups as the AE or PROMIS-29 outcomes could not be aligned with either product. This study only included patients taking oral liquid or capsule formulations to ensure consistency when analyzing dose and effectiveness, as inhaled formulations have variable absorption properties.^16^ Participants also reported the quantity (mL) they were prescribed, so the total dose of THC and/or CBD the participant consumed each day could be calculated (mg/day).

### Patient Outcomes

The effectiveness of medicinal cannabis for anxiety disorders was measured using the PROMIS-29 (v2.0) survey; a health-related quality of life (HRQoL) tool that evaluates health outcomes. Of the 7 domains in the survey, excessive anxiety, fatigue, sleep disturbance, and a decreased ability to take part in social roles and activities and depression were included in our analysis.^17,18^

Participants’ raw scores across these 5 health domains from each of their questionnaires were converted into t-scores using the PROMIS-29 v2.0 conversion tables. The difference between the scores of their baseline survey and final survey was calculated to analyze the outcomes after treatment with medicinal cannabis. Outcomes across each domain were assessed for clinical significance based on published minimal clinically important difference (MCID) scores. The MCID for the anxiety domain was set to 4,^19^ while the rest of the domains were set to 5 (depression, fatigue, pain impact, and sleep disturbance).^20^ Participants were classified as clinically “improved,” “unchanged,” or “worsened” based on a respective t-score change in relation to MCID.

### Adverse Events

Participants were able to self-report any AEs they experienced during their treatment with medicinal cannabis in the questionnaires. Common AEs were listed, and participants could tick to indicate what they had experienced. There was also a section where they could write freely anything they experienced. Each AE was then categorized into an organ class based on the Medical Dictionary for Regulatory Activities (MedDRA) System Organ Classes and reported by formulation type.^21^

### Statistical Analysis

A statistical analysis of the data was performed using Statistical Package for the Social Sciences (SPSS; IBM, Armonk, NY). Data for continuous variables, such as dose, duration of treatment, and participant age, were assessed for normality. Where a normal distribution for continuous variable data was observed, the mean and standard deviation were reported, but for data that were not normally distributed, the median and interquartile range (IQR) were reported.^22^ Paired 2-tailed *t* tests were performed to compare participant t-scores before and after taking medicinal cannabis across each PROMIS-29 domain to determine the significance of these results. A 1-way analysis of variance (ANOVA) was conducted to determine whether there were significant differences in t-score changes between formulation types, and a 2-way ANOVA^23^ was also used to compare the differences in t-score changes between both formulation categories and different types of anxiety diagnoses.

Fisher’s exact tests were used to compare the clinical improvement categorical results of “improved,” “unchanged,” and “worsened” across the PROMIS-29 domains for each participant subset, only including patients that had taken a single formulation type.^24^ If the clinical categorization for a PROMIS-29 domain was significant for a total participant subset, a further analysis was undertaken on the different formulation types.

Logistic regressions were performed to determine whether there was a relationship between the CBD/THC doses and clinical improvement in the PROMIS-29 domains or AE MedDRA classes. Where there was a significant association between the CBD/THC doses and AE class, a logistic regression was performed on the individual AEs in this class to determine where the significance was.

## Results

### Participant Demographics

From the CACOS study, 568 people were eligible for the AE analysis in this study, and 198 participants for the effectiveness (PROMIS-29) analysis. Demographic information of the participant sample is provided in Table 1.

**Table 1.** Demographics of Participants Included in This Study.

Demographic feature	Effectiveness analysis (n = 198)	Adverse events analysis (n = 568)
Sex		
Female, n (%)	105 (53.0)	304 (53.5)
Male, n (%)	93 (47.0)	264 (46.5)
Age, years, median (IQR)	48 (24)	48 (24)
Observational period, days, median (IQR)	154.4 (246.6)	55.8 (191.2)
Anxiety type		
PTSD, n (%)	57 (28.8)	158 (27.8)
Unspecified, n (%)	141 (71.2)	410 (72.2)

Abbreviations: IQR, interquartile range; PTSD, posttraumatic stress disorder.

### Patient Outcomes

There were statistically significant changes to participant outcomes observed in the total participant group (n = 198), PTSD subset (n = 57), and unspecified anxiety subset (n = 141) (Table 2). In the total participant sample (n = 198), 53% (n = 104) of participants were classified as having had a clinically meaningful improvement in their anxiety levels (a change in score greater than the MCID) (Table 2). Participants overall reported significantly improved anxiety (*P* < 0.001), depression (*P* < 0.001), fatigue (*P* < 0.001), and ability to take part in social roles and activities (*P* < 0.001). The CBD-only (*P* < 0.001), balanced (*P* < 0.001), and THC-dominant (*P* = 0.011) formulations were all associated with significant improvements in anxiety symptoms.

**Table 2.** Effectiveness (PROMIS-29) Analysis of all Participants With an Anxiety Diagnosis.

	All anxiety participants					
	All formulations (n = 198)	CBD-only (n = 112)	CBD-dominant (n = 20)	Balanced (n = 96)	THC-dominant (n = 18)	THC-only (n = 10)
CBD dose mg/day, median (IQR)	50.0 (85.0)	100.0 (100.0)	25.0 (43.5)	20.0 (40.0)	6.0 (13.6)	0.0 (0.0)
THC dose mg/day, median (IQR)	4.4 (20.0)	0.0 (0.0)	6.3 (25.7)	20.0 (34.3)	33.8 (61.5)	38.0 (35.7)
Anxiety (MCID = 4)						
Score baseline, mean (SD)	64.6 (9.0)	64.6 (8.8)	61.9 (11.3)	64.2 (8.9)	63.1 (8.5)	64.4 (8.0)
Score final, mean (SD)	59.6 (9.0)	60.2 (8.3)	58.9 (6.8)	59.9 (9.7)	58.2 (7.7)	62.4 (8.0)
*P* value	<0.001*	<0.001*	0.300	<0.001*	0.011*	0.587
Improved, n (%)	104 (52.5)	56 (50.0)	10 (50.0)	46 (47.9)	11 (61.1)	4 (40.0)
Unchanged, n (%)	66 (33.6)	38 (33.9)	5 (25.0)	36 (37.5)	5 (27.7)	3 (30.0)
Worsened, n (%)	28 (14.1)	18 (16.1)	4 (20.0)	14 (14.6)	2 (11.1)	3 (30.0)
*P* value**	0.945					
Depression (MCID = 5)						
Score baseline, mean (SD)	61.6 (9.8)	61.3 (9.8)	61.6 (9.5)	61.9 (10.4)	58.5 (10.6)	61.1 (10.2)
Score final, mean (SD)	57.5 (10.0)	58.3 (9.1)	58.3 (6.4)	57.9 (11.0)	53.9 (10.3)	59.3 (13.6)
*P* value	<0.001*	<0.001*	0.111	<0.001*	0.018*	0.713
Improved, n (%)	84 (42.4)	39 (34.8)	8 (40.0)	43 (44.8)	9 (50.0)	4 (40.0)
Unchanged, n (%)	86 (43.4)	59 (52.7)	8 (40.0)	33 (34.4)	7 (38.9)	2 (20.0)
Worsened, n (%)	28 (14.1)	14 (12.5)	4 (20.0)	20 (20.8)	2 (11.1)	4 (40.0)
*P* value**	0.032					
Fatigue (MCID = 5)						
Score baseline, mean (SD)	62.9 (10.0)	63.0 (9.2)	65.5 (8.5)	63.8 (10.4)	62.3 (12.5)	66.4 (9.4)
Score final, mean (SD)	56.9 (11.0)	56.1 (10.2)	58.6 (8.5)	58.2 (11.2)	55.5 (11.8)	57.4 (13.6)
*P* value	<0.001*	<0.001*	<0.001*	<0.001*	0.033*	0.014*
Improved, n (%)	95 (48.0)	52 (46.4)	10 (50.0)	46 (47.9)	10 (55.6)	8 (80.0)
Unchanged, n (%)	85 (42.9)	52 (46.4)	9 (45.0)	41 (42.7)	5 (27.8)	2 (20.0)
Worsened, n (%)	18 (9.1)	8 (7.1)	1 (5.0)	9 (9.4)	3 (16.7)	1 (10.0)
*P* value**	0.588					
Sleep disturbance (MCID = 5)						
Score baseline, mean (SD)	51.6 (5.4)	51.8 (4.1)	52.7 (3.4)	51.5 (6.7)	51.4 (5.8)	50.1 (6.9)
Score final, mean (SD)	51.6 (4.4)	51.7 (3.5)	52.0 (2.4)	51.60 (5.5)	51.4 (2.8)	52.6 (2.1)
*P* value	0.964	0.830	0.392	0.788	0.991	0.324
Improved, n (%)	26 (13.1)	11 (9.8)	4 (20.0)	16 (16.7)	4 (22.2)	2 (20.0)
Unchanged, n (%)	150 (75.8)	89 (79.5)	16 (80.0)	67 (69.8)	12 (66.7)	8 (80.0)
Worsened, n (%)	22 (11.1)	12 (10.7)	1 (5.0)	13 (13.5)	2 (11.1)	1 (10.0)
*P* value**	0.458					
Ability to take part in social roles and activities (MCID = 5)						
Score baseline, mean (SD)	36.5 (9.3)	37.3 (7.0)	36.1 (7.7)	34.9 (6.9)	37.7 (10.0)	34.7 (7.4)
Score final, mean (SD)	41.5 (9.7)	42.8 (9.2)	40.5 (11.3)	38.7 (9.3)	40.8 (10.4)	42.7 (14.6)
*P* value	<0.001*	<0.001*	0.024*	<0.001*	0.082	0.022*
Improved, n (%)	91 (46.0)	56 (50.0)	6 (30.0)	37 (38.5)	7 (38.9)	5 (50.0)
Unchanged, n (%)	91 (46.0)	49 (43.8)	13 (65.0)	49 (51.0)	9 (50.0)	5 (50.0)
Worsened, n (%)	16 (8.1)	7 (6.3)	1 (5.0)	10 (10.4)	2 (11.1)	0 (0.0)
*P* value**	0.642					

Abbreviations: CBD, cannabidiol; IQR, interquartile range; MCID, minimal clinically important difference; PROMIS-29, Patient-Reported Outcomes Measurement Information System-29; SD, standard deviation; THC, tetrahydrocannabinol.

* *P* value is statistically significant improvement in the symptom or condition (*P* < 0.05).

** *P* value calculated using Fisher’s exact tests including participants from the sample that had been on only 1 formulation.

Similar to the total participant sample, 52.6% (n = 30) of those diagnosed with PTSD reported a clinical improvement in anxiety symptoms, with statistical significance observed for anxiety (*P* < 0.001), depression (*P* < 0.001), fatigue (*P* < 0.001), and social abilities (*P* < 0.001). The CBD-only group (n = 35) was the only formulation associated with significant decreases in anxiety (*P* < 0.001) and depression (*P* = 0.019). Symptoms of depression were also significantly more likely to be categorized as clinically improved in participants taking CBD-only (*P* < 0.001) and balanced (*P* < 0.001) formulations. Participants with PTSD also reported significant improvements to their fatigue in the CBD-only (*P* < 0.001), balanced (*P* < 0.001), and THC-only groups (*P* = 0.009) and in their social ability while taking a CBD-only (*P* < 0.001) and balanced (*P* = 0.003) formulations.

The unspecified anxiety subset (n = 141) reported that anxiety symptoms significantly improved while taking the same medicinal cannabis formulations as the total patient sample. Participants in this cohort also reported significantly improved depression while taking CBD-only (*P* = 0.002) and balanced formulations (*P* = 0.006). Fatigue was significantly improved for those taking CBD-only (*P* < 0.001), CBD-dominant (*P* = 0.001), balanced (*P* < 0.001), and THC-dominant (*P* = 0.034) formulations. This was similar for social abilities, except for the THC-dominant formulation.

A 1-way ANOVA determined that there were no significant differences in health outcomes between formulation types, and a 2-way ANOVA confirmed that there were also no significant differences when factoring in the different anxiety disorder classifications. A logistic regression established there was no relationship between clinical improvement and CBD/THC dose in this participant sample.

### Adverse Events

The AEs experienced by participants were analyzed according to the formulation type(s) they had been prescribed throughout their observational period (Table 3). A total of 1314 AEs were reported across 568 participants. The maximum number of different AEs reported by a single participant across their total observational period was 13.

**Table 3.** Adverse Events Across Formulation Types by MedDRA System Organ Class.

MedDRA system organ class	All formulations (n = 568)		CBD-only (n = 297)		CBD-dominant (n = 75)		Balanced (n = 257)		THC-dominant (n = 51)		THC-only (n = 19)	
	AE^a^	n^b^	AE	n	AE	n	AE	n	AE	n	AE	n
CBD dose, median (IQR)	40.0 (87.6)		90 (109.1)		27 (46.3)		18.75 (21.3)		4.0 (7.1)		0.0 (0.4)	
THC dose, median (IQR)	5.0 (20.0)		0.0 (0.0)		8.0 (17.8)		19 (20.0)		30.0 (48.0)		33.0 (19.25)	
Gastro-intestinal disorders, n (%)												
Total	386 (29.4)	220 (38.7)	198 (29.4)	105 (35.4)	46 (21.0)	30 (40.0)	216 (29.7)	124 (48.2)	55 (26.6)	29 (56.9)	17 (23.9)	12 (63.2)
Dry mouth	274 (20.9)	185 (32.6)	140 (20.8)	87 (29.3)	30 (13.7)	23 (30.7)	152 (20.9)	104 (40.5)	41 (19.8)	25 (49.0)	15 (21.1)	10 (52.6)
Nausea	61 (4.6)	52 (9.2)	28 (4.2)	24 (8.1)	6 (2.7)	6 (8.0)	38 (5.2)	32 (12.5)	10 (4.8)	9 (17.6)	2 (2.8)	2 (10.5)
Diarrhea	20 (1.5)	19 (3.4)	9 (1.3)	9 (3.0)	3 (1.4)	2 (2.7)	13 (1.8)	12 (4.7)	2 (1.0)	2 (3.9)	0	0
Gastrointestinal upset	21 (1.6)	18 (3.2)	15 (2.2)	13 (4.4)	7 (3.2)	5 (6.7)	9 (1.2)	8 (3.1)	2 (1.0)	2 (3.9)	0	0
Vomiting	2 (0.2)	2 (0.4)	1 (0.1)	1 (0.3)	0	0	1 (0.1)	1 (0.4)	0	0	0	0
Constipation	4 (0.3)	3 (0.5)	4 (0.6)	3 (1.0)	0	0	0	0	0	0	0	0
Flatulence, bloating, and distension	4 (0.3)	2 (0.4)	1 (0.1)	1 (0.3)	0	0	3 (0.4)	1 (0.4)	0	0	0	0
Psychiatric, n (%)												
Total	498 (37.9)	233 (41.0)	261 (38.8)	119 (40.1)	82 (37.4)	32 (42.7)	271 (37.2)	121 (47.1)	60 (29.0)	29 (56.9)	28 (39.4)	12 (63.2)
Somnolence	239 (18.2)	178 (31.3)	120 (17.8)	88 (29.9)	40 (18.3)	26 (34.7)	136 (18.7)	96 (37.2)	31 (15.0)	24 (47.1)	13 (18.3)	9 (47.4)
Inappropriate laughter	2 (0.2)	2 (0.4)	1 (0.1)	1 (0.3)	0	0	2 (0.3)	2 (0.8)	0	0	0	0
Anxiety	71 (5.4)	54 (9.5)	44 (6.5)	34 (11.4)	10 (4.6)	9 (12.0)	30 (4.1)	23 (8.9)	6 (2.9)	4 (7.8)	3 (4.2)	1 (5.3)
Lack of motivation	1 (0.1)	1 (0.2)	0	0	1 (0.5)	1 (1.3)	0	0	0	0	0	0
Confusion	34 (2.6)	31 (5.5)	16 (2.4)	15 (5.1)	4 (1.8)	3 (4.0)	21 (2.9)	19 (7.4)	3 (1.4)	3 (5.9)	1 (1.4)	1 (5.3)
Disorientation	28 (2.1)	27 (4.8)	18 (2.7)	17 (5.7)	4 (1.8)	4 (5.3)	16 (2.2)	16 (6.2)	4 (1.9)	4 (7.8)	1 (1.4)	1 (5.3)
Depression	31 (2.4)	30 (5.5)	14 (2.1)	14 (4.7)	2 (0.9)	2 (2.7)	14 (1.9)	14 (5.4)	7 (3.4)	6 (11.8)	3 (4.2)	2 (10.5)
Paranoia	8 (0.6)	7 (1.2)	6 (0.9)	5 (1.7)	3 (1.4)	2 (2.7)	4 (0.6)	4 (1.6)	1 (0.5)	1 (2.0)	1 (1.4)	1 (5.3)
Euphoria	37 (2.8)	29 (5.1)	22 (3.3)	19 (6.4)	7 (3.2)	2 (2.7)	21 (2.9)	15 (3.8)	6 (2.9)	5 (9.8)	3 (4.2)	3 (15.8)
Hallucination	5 (0.4)	5 (0.9)	2 (0.3)	2 (0.7)	1 (0.5)	1 (1.3)	3 (0.4)	3 (1.2)	0	0	1 (1.4)	1 (5.3)
Insomnia	22 (1.7)	21 (3.7)	10 (1.5)	10 (3.4)	5 (2.3)	5 (6.7)	10 (1.4)	9 (3.5)	0	0	0	0
Psychosis	1 (0.1)	1 (0.2)	1 (0.1)	1 (0.3)	0	0	0	0	0	0	1 (1.4)	1 (5.3)
Cognitive impairment	1 (0.1)	1 (0.2)	0	0	1 (0.5)	1 (1.3)	1 (0.1)	1 (0.4)	0	0	0	0
Slowed thinking	2 (0.2)	1 (0.2)	2 (0.3)	1 (0.3)	0	0	2 (0.3)	1 (0.4)	0	0	0	0
Increased sex drive	1 (0.1)	1 (0.2)	0	0	0	0	1 (0.1)	1 (0.4)	0	0	0	0
Racing thoughts	1 (0.1)	1 (0.2)	0	0	0	0	1 (0.1)	1 (0.4)	0	0	0	0
Grinding teeth	1 (0.1)	1 (0.2)	0	0	0	0	1 (0.1)	1 (0.4)	0	0	0	0
Memory loss	9 (0.7)	8 (1.4)	2 (0.3)	2 (0.7)	4 (1.8)	3 (4.0)	6 (0.8)	5 (1.9)	2 (1.0)	2 (3.9)	0	0
Mood disorders and disturbances	4 (0.3)	4 (0.7)	3 (0.4)	3 (1.0)	0	0	2 (0.3)	2 (0.8)	0	0	1 (1.4)	1 (5.3)
Nervous system disorders, n (%)												
Total	95 (7.2)	73 (12.9)	53 (7.9)	36 (12.1)	19 (8.7)	15 (20.0)	49 (6.7)	38 (14.8)	16 (7.7)	9 (17.6)	3 (4.2)	3 (15.8)
Vivid dreams	1 (0.1)	1 (0.2)	1 (0.1)	1 (0.3)	0	0	0	0	0	0	0	0
Dizziness	69 (5.3)	62 (10.9)	35 (5.2)	29 (9.8)	13 (5.9)	13 (17.3)	35 (4.8)	33 (12.8)	10 (4.8)	9 (17.6)	3 (4.2)	3 (15.8)
Agitation	5 (0.4)	1 (0.2)	5 (0.7)	1 (0.3)	0	0	5 (0.7)	1 (0.4)	5 (2.4)	1 (2.0)	0	0
Tingling feeling	3 (0.2)	2 (0.4)	1 (0.1)	1 (0.3)	0	0	3 (0.4)	2 (0.8)	0	0	0	0
Tremor	1 (0.1)	1 (0.2)	0	0	0	0	1 (0.1)	1 (0.4)	0	0	0	0
Headache	16 (1.2)	13 (2.3)	11 (1.6)	8 (2.7)	6 (2.7)	3 (4.0)	5 (0.7)	5 (1.9)	1 (0.5)	1 (2.0)	0	0
Metabolism disorders, n (%)												
Total	79 (6.0)	62 (10.9)	37 (5.5)	30 (10.1)	18 (8.2)	14 (18.7)	47 (6.4)	35 (13.6)	14 (6.8)	8 (15.7)	6 (8.5)	3 (15.8)
Increase appetite	60 (4.6)	45 (7.9)	28 (4.2)	22 (7.4)	9 (4.1)	7 (9.3)	37 (5.1)	27 (10.5)	12 (5.8)	7 (13.7)	6 (8.5)	3 (15.8)
Decreased appetite	19 (1.5)	18 (3.2)	9 (1.3)	8 (2.7)	9 (4.1)	8 (10.7)	9 (1.2)	9 (3.5)	2 (1.0)	2 (3.9)	0	0
Skin disorders, n (%)												
Total	2 (0.2)	2 (0.4)	1 (0.1)	1 (0.3)	0	0	0	0	1 (0.5)	1 (2.0)	0	0
Acne	1 (0.1)	1 (0.2)	1 (0.1)	1 (0.3)	0	0	0	0	0	0	0	0
Skin irritation	1 (0.1)	1 (0.2)	0	0	0	0	0	0	1 (0.5)	1 (2.0)	0	0
General disorders and administration site conditions, n (%)												
Total	197 (15.0)	131 (23.1)	90 (13.4)	62 (20.9)	43 (19.6)	25 (32.3)	108 (15.0)	69 (26.8)	38 (18.4)	19 (37.3)	10 (14.1)	4 (21.1)
Fatigue	125 (9.5)	105 (18.5)	56 (8.3)	48 (16.2)	23 (10.5)	20 (26.7)	70 (9.6)	58 (22.6)	22 (10.6)	17 (33.3)	4 (5.6)	4 (21.1)
Balance problems	48 (3.7)	40 (7.0)	21 (3.1)	17 (5.7)	7 (3.2)	7 (9.3)	24 (3.3)	21 (8.2)	8 (3.9)	6 (11.8)	2 (2.8)	2 (10.5)
Foggy feeling in head	2 (0.2)	2 (0.4)	1 (0.1)	1 (0.3)	0	0	1 (0.1)	1 (0.4)	0	0	0	0
Feeling of relaxation	4 (0.3)	1 (0.2)	0	0	4 (1.8)	1 (1.3)	4 (0.6)	1 (0.4)	0	0	0	0
Weight on chest	1 (0.1)	1 (0.2)	1 (0.1)	1 (0.3)	0	0	0	0	0	0	0	0
Energy increased	2 (0.2)	2 (0.4)	2 (0.3)	2 (0.7)	1 (0.5)	1 (1.3)	0	0	0	0	0	0
Increased thirst	1 (0.1)	1 (0.2)	0	0	1 (0.5)	1 (1.3)	1 (0.1)	1 (0.4)	0	0	0	0
Spaced out feeling	4 (0.3)	1 (0.2)	0	0	4 (1.8)	1 (1.3)	4 (0.6)	1 (0.4)	4 (1.9)	1 (2.0)	4 (5.6)	1 (5.3)
Pain	10 (0.8)	6 (1.1)	9 (1.3)	5 (1.7)	3 (1.4)	1 (1.3)	4 (0.6)	2 (0.8)	4 (1.9)	2 (3.9)	0	0
Eye disorders, n (%)												
Total	19 (1.5)	5 (0.9)	13 (1.9)	2 (0.7)	6 (2.7)	3 (4.0)	14 (1.9)	3 (1.2)	13 (6.3)	2 (3.9)	4 (5.6)	1 (5.3)
Vision issues	4 (0.3)	2 (0.4)	3 (0.5)	1 (0.3)	4 (1.8)	2 (2.7)	0	0	0	0	0	0
Dry eyes	15 (1.1)	4 (0.7)	10 (1.5)	2 (0.7)	2 (0.9)	2 (2.7)	14 (1.9)	3 (1.2)	13 (6.3)	2 (3.9)	4 (5.6)	1 (5.3)
Respiratory thoracic and mediastinal disorders, n (%)												
Total	6 (0.5)	6 (1.1)	5 (0.7)	5 (1.7)	2 (0.9)	2 (2.7)	3 (0.4)	3 (1.2)	1 (0.5)	1 (2.0)	0	0
Sore throat	6 (0.5)	6 (1.1)	5 (0.7)	5 (1.7)	2 (0.9)	2 (2.7)	3 (0.4)	3 (1.2)	1 (0.5)	1 (2.0)	0	0
Cardiac disorders, n (%)												
Total	5 (0.4)	3 (0.5)	2 (0.3)	2 (0.7)	0	0	2 (0.3)	1 (0.4)	0	0	0	0
Arrhythmia	2 (0.2)	2 (0.4)	2 (0.3)	2 (0.7)	0	0	0	0	0	0	0	0
Palpitations	3 (0.2)	1 (0.2)	0	0	0	0	3 (0.4)	1 (0.4)	0)	0	0	0
Musculoskeletal and connective tissue disorders, n (%)												
Total	5 (0.4)	4 (0.7)	0	0	0	0	4 (0.6)	3 (1.2)	1 (0.5)	1 (2.0)	0	0
Muscle twitching	1 (0.1)	1 (0.2)	0	0	0	0	1 (0.1)	1 (0.4)	0	0	0	0
Mobility decreased	1 (0.1)	1 (0.2)	0	0	0	0	1 (0.1)	1 (0.4)	0	0	0	0
Muscle tension	2 (0.2)	1 (0.2)	0	0	0	0	2 (0.3)	1 (0.4)	0	0	0	0
Swollen ankles	1 (0.1)	1 (0.2)	0	0	0	0	0	0	1 (0.5)	1 (2.0)	0	0
Ear and labyrinth disorders, n (%)												
Total	1 (0.1)	1 (0.2)	1 (0.1)	1 (0.3)	0	0	1 (0.1)	1 (0.4)	0	0	0	0
Tinnitus	1 (0.1)	1 (0.2)	1 (0.1)	1 (0.3)	0	0	1 (0.1)	1 (0.4)	0	0	0	0
Renal and urinary disorders, n (%)												
Total	1 (0.1)	1 (0.2)	0	0	0	0	1 (0.1)	1 (0.4)	0	0	0	0
Increased urination	1 (0.1)	1 (0.2)	0	0	0	0	1 (0.1)	1 (0.4)	0	0	0	0
Immune system disorders, n (%)												
Total	2 (0.2)	1 (0.2)	2 (0.3)	1 (0.3)	0	0	0	0	0	0	0	0
Allergy	2 (0.2)	1 (0.2)	2 (0.3)	1 (0.3)	0	0	0	0	0	0	0	0
Other (undefined), n (%)	18 (1.4)	15 (2.6)	10 (1.5)	7 (2.4)	3 (1.4)	3 (4.0)	11 (1.5)	9 (3.5)	8 (3.7)	6 (11.8)	3 (4.2)	3 (15.8)
Total n	1314	568	673	297	219	75	727	257	207	51	71	19
Number of patients that never reported AEs, n (%)	227 (40.0)		120 (40.4)		29 (38.7)		89 (34.6)		13 (25.5)		3 (15.8)	

Abbreviations: AE, adverse events; CBD, cannabidiol; IQR, interquartile range; MedDRA, Medical Dictionary for Regulatory Activities; THC, tetrahydrocannabinol.

a AE refers to total number of AEs reported.

b n Refers to total number of participants that reported the AE.

There were 227 (40.0%) participants that never reported an AE, and the CBD-only group had the greatest proportion of participants who were in this category (n = 120, 40.4%). The most common type of AE recorded across all formulation types was the psychiatric system organ class (n = 233, 41.0%). The most common psychiatric AEs experienced by participants were somnolence (31.3%), anxiety (9.5%), and euphoria (5.1%). Where participants reported anxiety as an AE, it indicated they experienced increased anxiety symptoms since commencing treatment with medicinal cannabis.

Other common AEs included dry mouth (n = 185, 32.6%), fatigue (n = 105, 18.5%), and dizziness (n = 62, 10.9%). A logistic regression established a relationship between THC concentration and gastrointestinal AEs (odds ratio [OR] = 1.011, *P* = 0.003), specifically dry mouth (OR = 1.010, *P* = 0.005) and nausea (OR = 1.008, *P* = 0.008); however, these are unlikely to be clinically significant with an OR close to 1. There was no significant association between reporting anxiety and CBD and/or THC doses.

In participants taking CBD-only formulations, somnolence and dry mouth were the most common AEs, reported by 88 (29.9%) and 87 (29.3%) participants, respectively. The THC-only subset had the highest proportion of participants who reported euphoria (n = 3, 15.8%), followed by the THC-dominant formulation group (n = 5, 9.8%).

## Discussion

This observational, retrospective study of participants using medicinal cannabis for anxiety disorders analyzed the effectiveness of medicinal cannabis on different HRQoL outcomes. Participants taking the CBD-only and balanced formulations reported improved levels of anxiety, depression, fatigue, and ability to participate in social activity in both the full participant sample and the unspecified anxiety subset. In the PTSD participant subset, the CBD-only formulation group at a median (IQR) dose of 95 (117.6) mg/day was the only group that reported significant improvements in the same 4 participant outcomes.

This study provides important insights into the current practice of prescribing medicinal cannabis for anxiety conditions and the type and incidence of AEs that the participants experienced. It is important to note, the research was limited as it relied on data gathered from surveys that participants completed themselves, and therefore, recall bias and misclassification bias were not accounted for. This study cohort may also be subject to potential sample bias as the characteristics of patients who chose not to enroll in CACOS are not known. Future studies should attempt to study participants across multiple sites and with diverse characteristics to increase external validity. As this study was observational, it can only establish that medicinal cannabis is associated with the identified outcomes, and therefore, it cannot be concluded that medicinal cannabis is the cause of these outcomes. Some participants were switched between medicinal cannabis formulations during the observational period and were included in both groups for analysis. This was done as the AE or PROMIS-29 outcomes could not be aligned with either product, potentially limiting our results. Future, randomized, placebo-controlled studies are imperative to determine a causal relationship between the individual medicinal cannabis formulations and effectiveness and AE outcomes. The AE analysis did not account for the number of surveys a single participant completed, the severity of effect, or if they were collected less than 7 days apart unlike the PROMIS analysis. This study did not analyze the dose and AEs, meaning it could not determine whether there was an association between higher doses and a greater incidence of AEs. Future research should address characteristics that may affect whether participants find CBD and/or THC effective and at what doses. With the use of medicinal cannabis increasing before there is robust evidence from randomized controlled trials, real-world evidence such as this is of increasing value and will be important in informing the controlled studies.

From this analysis, CBD-only formulations are associated with the most-improved outcomes in participants with PTSD; however, this study only used a small group of PTSD participants, so future studies with more participants on other medicinal cannabis formulations are needed. The effectiveness of CBD-only formulations for PTSD was also observed in a case series of 11 participants that were treated with CBD (48.6 mg/day median start dose) as an adjunct to concurrent psychiatric medications.^25^ A decrease in PTSD symptoms, as measured by the PTSD Checklist for Diagnostic Statistical Manual of Mental Disorders, was reported for 91% (n = 10) of the participants in our study; this suggests that lower doses of CBD could be as effective as higher doses.^26^ It is important to note that concurrent psychiatric medications were reported but not accounted for or analyzed in the case series and not reported in our study.

Participants who took a THC-dominant formulation reported a significant decrease in their anxiety levels. Those same participants also represented the highest proportion of participants that were classified as having clinical improvement (61.1%, n = 11), compared with participants who were prescribed other formulation types. This was unexpected as the median THC dose for this participant group was 33.8 mg/day, and it has been suggested that doses higher than 30 mg/day could be anxiogenic.^14^ A study reported that doses of CBD ranging from 15 to 60 mg/day could offset the anxiogenic properties of THC, which is reflected in our data; however, with lower doses of CBD (median = 6.0 mg/day CBD).^14^

Self-reported participant anxiety symptoms significantly improved in participants with unspecified anxiety that used CBD-only, balanced, and THC formulations. In Australia, the most common types of anxiety disorders are PTSD (6.4%), followed by social anxiety (4.7%) and generalized anxiety disorder (2.7%).^26^ Due to the significant prevalence of social anxiety in Australia, it can be assumed that a portion of the participants in the undiagnosed anxiety group had a social anxiety diagnosis. Similar to our results, a study on CBD for participants with social anxiety found that there were significant improvements to participant anxiety levels when measured by both the Fear of Negative Evaluation Questionnaire and the Liebowitz Social Anxiety Scale measurements.^27^ Another study that looked at participants with an unspecified anxiety diagnosis who took CBD capsules 25 to 175 mg/day reported improved anxiety scores for 79% of the participants (n = 57).^28^ These results are not directly comparable to our results for the CBD-only participants, as we reported the number of participants that were classified as clinically improved (n = 38, 49.4%).

There were significant improvements in the depression outcomes for participants taking CBD-only, balanced, and THC-dominant formulations. For PTSD participants, CBD-only and balanced formulations were also found to have a significant association with clinically improved depression outcomes. There was no association between the CBD/THC dose and participant improvement in any of the 5 health outcomes. As such, further studies will need to be conducted to determine the optimal dose for PTSD and other anxiety conditions.

Overall, we found that 60% of participants (n = 341) reported at least 1 AE. The most common AEs reported in this study were dry mouth, somnolence, fatigue, and dizziness; consistent with other studies.^29^ An Australian study that recruited 1302 participants who had been taking medicinal cannabis and reported AEs found that the most common AE was increased appetite, followed by somnolence, ocular irritation, and a lack of energy.^30^ The incidence of AEs was much higher in this sample; however, the most common AEs were similar to those observed in our study.

## Conclusions

In this study, participants taking medicinal cannabis reported significantly improved participant-reported outcomes of anxiety, depression, fatigue, and the ability to participate in social activities for participants with anxiety disorders. The only formulation type to improve all 4 of these participant outcomes in the total participant sample and subsets of unspecified anxiety and PTSD was CBD-only. The most common AEs experienced by participants were dry mouth, somnolence, and fatigue. There were significant associations between the THC dose and gastrointestinal AEs such as dry mouth and nausea. Further studies into medicinal cannabis should aim to establish the optimal dose and dosage regimen of CBD/THC for participants with anxiety disorders. A more specific analysis should also be undertaken into the effectiveness of medicinal cannabis for other anxiety disorders such as social anxiety and generalized anxiety disorder.
